# Marker-trait associations in two-rowed spring barley accessions from Kazakhstan and the USA

**DOI:** 10.1371/journal.pone.0205421

**Published:** 2018-10-11

**Authors:** Yuliya Genievskaya, Shyryn Almerekova, Burabai Sariev, Vladimir Chudinov, Laura Tokhetova, Grigoriy Sereda, Anarbai Ortaev, Vladimir Tsygankov, Thomas Blake, Shiaoman Chao, Kazuhiro Sato, Saule Abugalieva, Yerlan Turuspekov

**Affiliations:** 1 Institute of Plant Biology and Biotechnology, Almaty, Kazakhstan; 2 al-Farabi Kazakh National University, Department of Biodiversity and Bioresources, Almaty, Kazakhstan; 3 Kazakh Research Institute of Agriculture and Plant Industry, Almaty region, Kazakhstan; 4 Karabalyk Breeding Station, Kostanai region, Kazakhstan; 5 Kazakh Research Institute of Rice, Kyzylorda, Kazakhstan; 6 Karaganda Breeding Station, Karaganda region, Kazakhstan; 7 Krasnovodopad Breeding Station, South Kazakhstan region, Kazakhstan; 8 Aktobe Breeding Station, Aktobe region, Kazakhstan; 9 Department of Plant Sciences and Plant Pathology, Montana State University, Bozeman, MT, United States of America; 10 USDA-ARS Biosciences Research Lab, Fargo, ND, United States of America; 11 Research Institute for Bioresources, Okayama University, Kurashiki, Japan; Julius Kühn-Institut, GERMANY

## Abstract

In this study, phenotyping and single nucleotide polymorphism (SNP) genotyping data of 272 accessions of two-rowed spring barley from the USA along with 94 accessions from Kazakhstan were assessed in field trials at six breeding organizations in Kazakhstan to evaluate the performance of the USA samples over three years (2009–2011). The average grain yield over the six locations was not significantly higher in Kazakh accessions in comparison to the USA samples. Twenty four samples from Montana, Washington, the USDA station in Aberdeen Idaho, and the Anheuser-Busch breeding programs showed heavier average yield than the local standard cultivar “Ubagan”. Principal Coordinate analysis based on two sets of SNP data suggested that Kazakh accessions were closest to the USA accessions among eight groups of samples from different parts of the World, and within five US barley origin groups the samples from Montana and Washington perfectly matched six groups of Kazakh breeding origins. A genome-wide association study (GWAS) using data from eighteen field trials allowed the identification of ninety one marker-trait associations (MTA) in two or more environments for nine traits, including key characters such as heading time (HT), number of kernels per spike (NKS), and thousand grain weight (TGW). Our GWAS allowed the identification of eight MTA for HT and NKS, and sixteen MTA for TGW, when those MTA were linked to mapped SNPs. Based on comparisons of chromosomal positions of MTA identified in this study, and positions of known genes and quantitative trait loci for HT, NKS and TGW, it was suggested that MTA for HT on chromosome 2H (at 158.2 cM, 11_21414), MTA for NKS on 5H (at 118.6 cM, 11_20298), and two MTA for TGW on chromosome 4H (at 94.7 cM, 12_30718, and at 129.3 cM, 11_20013) were potentially new associations in barley. GWAS suggested that six MTA for HT, including two on chromosome 1H, two on chromosome 3H, and one each on chromosomes 4H and 6H, had useful pleiotropic effects for improving barley spike traits.

## Introduction

Barley (*Hordeum vulgare* L. spp *vulgare*) is an important crop in the agricultural sector of Kazakhstan, and it is grown in many different climatic zones over 1.5 million hectares annually. Currently, it is the second most widely-grown cereal crop in the country after wheat with on average an annual total grain yield of 2.0 million tons [1]. The end use for barley in the country is animal feed, and the average yield is 1.5 ton per hectare [1]. Traditionally, two-rowed spring barley is the dominant type in all major barley growing regions as the country has long and cold winters and often arid summers. The summertime is stressful in two out of three years due to drought and heat causing substantial grain yield loss [1].

As barley is cultivated in a wide range of Kazakh environments, it is important to develop a discrete breeding program for each of those regions to achieve highest possible grain yield and grain quality. One of the ways of improving the efficiency of regional breeding projects is the introduction of foreign germplasm from countries with similar environmental conditions [2, 3]. Historically, barley breeding programs in Kazakhstan were strongly connected to breeding organizations in the Russian Federation, as these two neighboring countries share a long boundary and similar climate conditions with the adjacent Siberian regions [4]. Also, since Kazakhstan was part of the former Soviet Union, both countries actively exchanged barley genetic resources [4, 5]. Currently 13 cultivars from Russia have been registered through the State Seed Trials Commission of the Republic of Kazakhstan [6]. It seems that potential sources of germplasm for barley breeding activities in Kazakhstan can come from countries with similar environments in terms of climate and latitude, such as the USA. As most of the USA barley breeding organizations were previously unified in the Barley Coordinated Agricultural Program (CAP) (that later was transformed to a Triticeae CAP), large barley resources, including germplasm, were generated [7, 8, 9], and can be successfully used in breeding projects around the World.

The other way to improve the efficiency of breeding programs is by the incorporation of modern genomic technologies [10, 11, 12]. In particularly, the automated genome-wide profiling of many agricultural crops, including barley [13, 14, 15, 16], with single nucleotide polymorphism (SNP) markers, is increasingly applied for the evaluation of genetic resources. In recent years, an Illumina-based SNP genotyping platform was successfully used both for the evaluation of wild [17, 18, 19] and cultivated barley accessions [20, 21, 22, 23] around the World. This trend was particularly important for the genetic mapping of quantitative trait loci (QTL) of agronomic traits based on the development of genome-wide association studies (GWAS). In barley there are several reports demonstrating the high efficiency of GWAS in the identification of marker-trait associations (MTA) for quantitative traits associated with morphological characters [24], abiotic stress tolerance [25], disease resistance [21, 26], and grain quality [27].

A survey of the published GWAS articles for cereal crops, including barley, is suggesting the strong influence of the growth environment on detection of QTL for yield components [28, 29, 30]. This can be explained by the sensitivity to environmental factors at flowering time and time to seed maturation that determine the potential number of grains per ear, as well as other yield components [31].

Thus, the success of national projects may largely depend on carrying out regional GWAS performed using both local and foreign germplasm. The main goal of this work was GWAS using spring two-rowed barley accessions from Kazakhstan and the USA for the identification of MTA in field trials in six diverse environments of Kazakhstan, and thus enhancing the efficiency of spring barley breeding projects in the country.

## Materials and methods

### Plant material

The collection of germplasm studied consisted of 366 accessions of two-rowed spring barley cultivars (n = 35) and breeding lines (n = 331) (*Hordeum vulgare* L. spp *vulgare*). The first group of the collection included 94 cultivars and promising lines provided by six breeding organizations of Kazakhstan (S1 Table) and represented the majority of local genetic pool of barley. The list of organizations providing their cultivars and lines were Karabalyk breeding station (North Kazakhstan, KB), Karaganda breeding station (Central Kazakhstan, KA), Aktobe breeding station (West Kazakhstan, AK), Almaty breeding station (South-east Kazakhstan, AL), Kazakh Research Institute of Rice (Kyzylorda city, South Kazakhstan, KO), and Krasnovodopad breeding station (South Kazakhstan, KV). Cultivar Ubagan was used as a standard for comparative field studies in the Northern, Central and Western regions, and cultivar Arna as a standard for Southern and South-eastern regions. The second part of the collection consisted of 272 accessions of barley (268 breeding lines and 4 cultivars) from the USA Barley Coordinated Agricultural Project (CAP) (S2 Table). The seeds of the accessions from the USA were provided by Dr. T. Blake, Montana State University (Bozeman, MT, USA) and represented five USA breeding organizations, including Montana State University (MT), Washington State University (WA), Utah State University (UT), Small Cereal Collection of the USDA held in Aberdeen, Idaho (AB), and one private company, Busch Agricultural Resources, a division of the Anheuser-Busch Corporation (BA). Breeding lines from the two groups were represented by advanced lines from the breeding programs, and considered to be pure lines.

### Field evaluation of the collections

**Fig 1:**
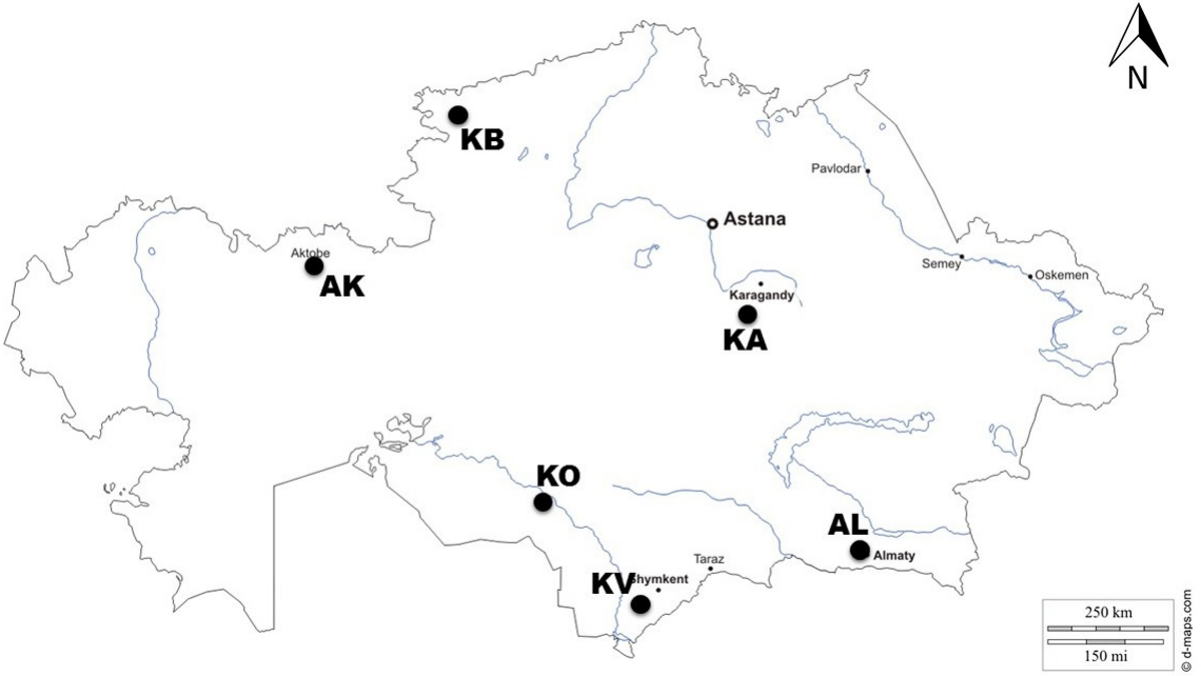
Locations of field trials at six breeding stations in Kazakhstan. KB–Karabalyk breeding station (North Kazakhstan), AK–Aktobe breeding station (West Kazakhstan), KA–Karaganda breeding station (Central Kazakhstan), KO–Kazakh Research Institute of Rice (Kyzylorda city, South Kazakhstan), KV–Krasnovodopad breeding station (South Kazakhstan), AL–Almaty breeding station (South-east Kazakhstan).

Phenotypic evaluations for the accessions were carried out in the experimental fields of the six major breeding institutions of Kazakhstan representing five regions–West, North, Center, South and South-east (Fig 1) over the years 2009–2011. In each year, each line was grown in three replicated one meter plots at each site. In total, 10 agronomic traits connected with flowering, plant architecture, and yield components were studied: days to heading (HT), days to seed maturation (SMT), plant height (PH), peduncle length (PL), productive tillering (PT), spike length (SL), number of kernels per spike (NKS), rachis internode length (RIL), thousand grain weight (TGW), and yield per square meter (YM2). Evaluation protocols for each trait were standardized for all breeding organizations participating in this study, and measured according to Ren et al. (2013) [32], except that RIL was calculated as spike length (mm) divided by the number of fertile rachis nodes [33]. The mean values of the 10 agronomic traits of the 366 two-rowed spring barley accessions harvested in six environments were subjected to further statistical analysis. Except KO, all remaining five locations performed field trials under non-irrigated conditions.

To assess genotype x environment interaction (GEI) patterns for plant adaptation traits and yield components with respect to location, the data were averaged over the three years at each location, to avoid unpredictable environmental fluctuations from year to year. The weather conditions in experimental fields, including temperature, precipitation, photoperiod lengths and soil types, were collected and provided by breeding institutions (S1 Fig). Statistical analyses of field data of the collection were evaluated using GraphPad Prism version 5.04 (GraphPad Software, La Jolla California USA. www.graphpad.com) and Statistika version 12.0 (StatSoft, Inc., 2013. http://www.statsoft.com/textbook/) GGE (genotype main effect and genotype × environment interaction) biplot graphics were developed with GenStat software 18^th^ ed. (VSN International Ltd, 2011. www.GenStat.co.uk). GGE plots were developed by using normalized data in symmetric scale.

### Genotyping of the collection

Ninety four accessions from Kazakhstan were genotyped using the GoldenGate Illumina 9K SNP chip at the TraitGenetics company (TraitGenetics GmbH, Gatersleben, Germany). The SNP genotyping data of BOPA1 and BOPA2 (Barley Oligo Pool Assay) sets of Illumina assays [14] of the USA accessions was provided by Dr. T. Blake, through the Triticeae toolbox (www.triticeaetoolbox.org). These two sets of data were merged to have 3072 SNP markers for both Kazakhstan and USA accessions. The total number of markers obtained was processed using the criteria as described by Miyagawa et al. [34]. These criteria include removing all monomorphic markers, markers with the call rate of SNP < 0.95 and MAF (minor allele frequency) < 0.05. As a result, 2321 polymorphic markers satisfied the set criteria and were used for further analysis. An additional set of samples consisting of 166 accessions representing eight regions of the World (S3 Table), including Kazakhstan, was genotyped using the 9K SNP Illumina genotyping assay. PCR, hybridization, and scanning for these 166 accessions were performed according to the Illumina genotyping assay protocol [35] at the Institute of Plant Science and Resources, Okayama University, Japan. SNP base calling was performed using GenomeStudio software version V2011.1 (Illumina Inc., 2018. http://jp.support.illumina.com).

### Association analysis

Analysis of the population structure was performed using the program STRUCTURE v2.3.4 with a Bayesian Markov Chain Monte Carlo (MCMC) approach based on admixture and correlated allele frequency models [36, 37]. The K value was set from 1 to 10; burnin period was set to 100000 and the number of MCMC replications after each burnin to 100000. The iteration number was 5. The ΔK values were visualized using the STRUCTURE HARVESTER v0.6.94 web-based program [38]. Based on the detection of optimal K value, the membership coefficient matrix of individuals (Q-matrix) was obtained in order to estimate the relatedness of each genotype to each group of samples.

The principal coordinate analysis (PCoA) was performed for the relationship analyses of accessions with different origins. Linkage disequilibrium (LD, r2) analysis was done using the 2321 polymorphic SNP markers dataset. The Kinship matrix and LD data were developed using TASSEL 5.0 [39]. The statistical software R was used for the visualization of the LD decay plot. The GWAS was based on using TASSEL 5.0 [39] and the Mixed Linear Model (MLM) [40, 41] using Kinship (K) and Q matrices. The significant associations were selected after application of a threshold bar at P<10E-4. To confirm the correction due to both K and Q matrices usage, the distribution lines in each of the quintile-quintile (QQ) plots were analyzed. Genetic maps were drawn using SNP locations in Muñoz-Amatriaín et al., 2014 [15].

## Results

### Comparative field performance of Kazakh and US accessions and GEI patterns

**Fig 2:**
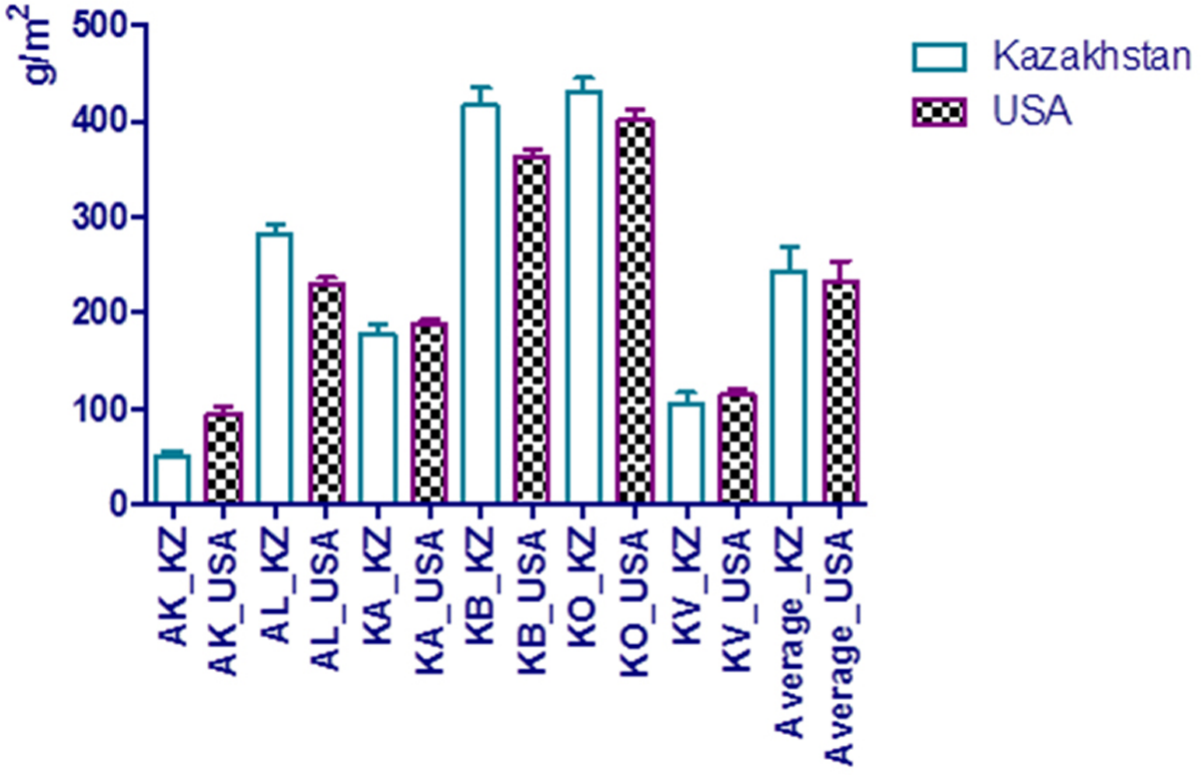
Average grain yield performance (g/m2) of USA and Kazakhstan barley groups at six field stations in Kazakhstan. KB–Karabalyk breeding station (North Kazakhstan), AK–Aktobe breeding station (West Kazakhstan), KA–Karaganda breeding station (Central Kazakhstan), KO–Kazakh Research Institute of Rice (Kyzylorda city, South Kazakhstan), KV–Krasnovodopad breeding station (South Kazakhstan), AL–Almaty breeding station (South-east Kazakhstan).

**Fig 3:**
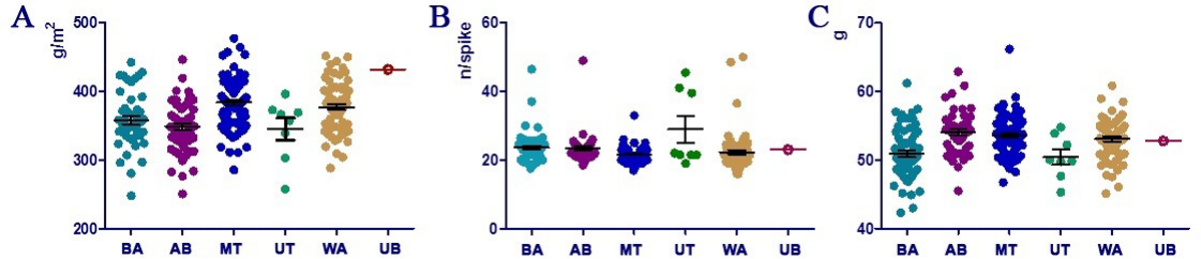
Comparisons between barley accessions from five breeding institutions of the USA and the standard Kazakh cultivar Ubagan (UB) at the Karabalyk breeding station, Northern Kazakhstan. Bars denote 95% confidence intervals. MT–Montana State University, WA–Washington State University, UT–Utah State University, AB–Small Cereal Collection of the USDA held in Aberdeen (Idaho), BA–Busch Agricultural Resources, a division of the Anheuser-Busch Corporation. A. Grain yield per m2 (YM2). B. Number of kernels per spike (NKS). C. Thousand grain weight (TGW).

Three-way analysis of variance (ANOVA) suggested highly significant differences (P<0.0001) for all the traits studied across all environments, and revealed significant effects of environment, genotype, and strong GEI (S4 Table). Averaged YM2 data for all 366 accessions over three years suggested that the KO and KB sites were the locations that were highest yielding out of the six environments (Fig 2). When averaged, YM2 data over three years were analyzed for the six regions by the Pearson correlation method, only four significant correlations were found. While the correlation between KB and KO was positive (P<0.008), three remaining correlations were negative (Table 1). The analysis of the correlation between traits over eighteen environment/years suggested that YM2 is positively correlated with SMT and NKS, and negatively with PH (Table 2). The average YM2 values in the Kazakh and USA groups over the six regions were comparable, and not significantly higher for the local accessions (Fig 2). This trend was similar when individual accessions of both groups were compared for NKS and TGW values, including the critically important KB environments (Fig 3). The KB breeding station is located in Northern Kazakhstan, where barley is grown on 80% of the total acreage. Therefore, all individual accessions from the USA were compared with the local standard variety “Ubagan” using averaged YM2, NKS and TGW over all seasons (Fig 3). It was found that twenty four individual accessions from MT, WA, AB, and BA showed higher average YM2 than “Ubagan”, and even more samples outperformed for NKS (n = 78) and TGW (n = 133) (Fig 3).

**Table 1:** Pearson correlation index for averaged YM2 in six environments.

	AK	AL	KA	KB	KO
AK	.	.	.	.	.
AL	-0.640*	.	.	.	.
KA	0.301	-0.335	.	.	.
KB	-0.626*	0.257	0.342	.	.
KO	-0.565	0.075	0.042	0.715*	.
KV	0.344	0.179	-0.528	-0.746*	-0.478

*–P<0.05

**Table 2:** Pearson correlation index between traits over eighteen environments using averaged data for lines from six Kazakh and five USA breeding sources.

	HT	SMT	PH	RIL	NKS	TGW
HT	.	.	.	.	.	.
SMT	0.378*	.	.	.	.	.
PH	0.433*	-0.255	.	.	.	.
RIL	0.049	-0.118	0.231	.	.	.
NKS	0.683**	0.556*	0.236	-0.542*	.	.
TGW	0.661**	0.760***	0.429*	0.706*	-0.174	.
YM2	0.269	0.715***	-0.303*	-0.154	0.640**	0.050

*–P<0.05

**–P<0.001

***–P<0.0001

The traits are given in abbreviations and their full names provided in the text.

Averaged data over three years for three plant adaptation traits (HT, SMT, and PH) and three grain yield components (NKS, TGW, and YM2) were analyzed in six different environments using the GGE biplot method. In the analyses of these three plant adaptation traits, the total variation in plots ranged from 61.4% in SMT to 85.3% in HT, and Kazakh and USA groups of samples were separated in different ways for all three traits (S2 Fig). For HT, all US-origin accessions were located on the right, and Kazakh lines on the left side of the plot, and AB samples were best suited for all of environmental locations, except AL (South-east region). In the SMT analysis the majority of lines of US breeding origins were positioned separately from the Kazakh lines on the left lower part of the plot, and UT accessions were close to two South Kazakhstan locations (KV and KO). For PH, all lines of US origin were located on left part of the plot, and those of AD origin were near the AK location that was separated from the remaining five environments (S2 Fig).

A slightly different outcome was observed in the GGE biplot analyses for the three yield components, NKS, TGW, and YM2 (S3 Fig). While for YM2 the USA lines grouped in the upper left side and well separated from those of Kazakh origin, for NKS and TGW the difference was not distinct. In the NKS plot the UT origin accessions were detached from the rest of the US samples and located close to most of the Kazakh environments, while the TGW plot showed that KB accessions were, as expected, favored for the KB environment, and KV samples for the remaining five locations (S3 Fig).

### Genetic diversity and population structure analyses

The genotyping of Kazakhstan and USA accessions allowed the identification of 2135 common polymorphic SNP markers distributed over all seven chromosomes with an average spacing of 1.33 cM. The number of SNP per chromosome ranged from 242 on chromosome 1H to 397 on chromosome 5H. The data also included 186 SNPs with unknown (U) positions. Additional information for each individual U marker can be retrieved from Muñoz-Amatriaín et al., 2014 [15] and the physical map Morex x Barke, 2016 [42]. The chromosomal length varied from 123.29 cM in chromosome 4H to 196.85 cM in chromosome 5H, with the average distance being 155.52 cM per chromosome. Polymorphism information content (PIC) values varied between 0.27 (1H and 4H) and 0.33 (5H).

**Fig 4:**
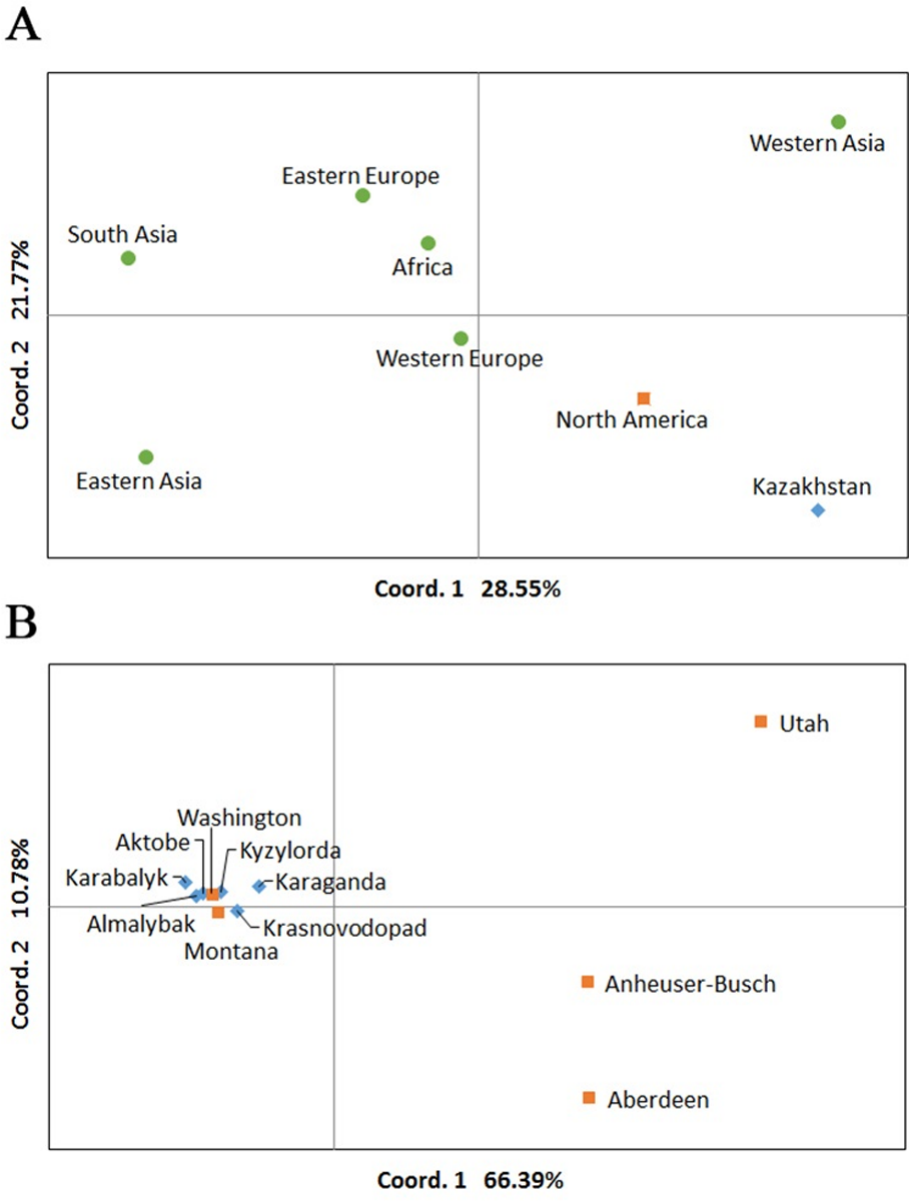
Principal coordinate analysis of barley accession SNP data. A. Clustering of barley accessions from eight regions of the World based on using the 9 K SNP Illumina genotyping assay. B. Clustering of accessions from five USA and six Kazakh breeding organizations based on 2321 polymorphic SNPs from the two Barley Oligo Pool Assay (BOPA1 and BOPA2) sets [14].

PCoA was applied to analyze the genetic relationships in the two sets of data. The first set included 166 accessions from eight regions of the World, including 96 samples from Kazakhstan. The PCoA plot suggested that samples from Kazakhstan were well separated from others and the closest group of accessions was from Northern America (Fig 4A), consisting of nineteen USA and one Canadian accessions. The second set consisted from 366 accessions divided into six Kazakhstan and five USA groups according to their breeding origin, genotyped by 2135 polymorphic SNP markers. PCoA showed that genotypes from Washington and Montana (USA) were genetically close to genotypes from all breeding origins in Kazakhstan (Fig 4B).

Population structures of the Kazakhstan and USA accessions were calculated to assess the structural pattern of the collection. Results obtained via STRUCTURE showed obvious partitioning of accessions into sub-clusters within the population. The STRUCTURE HARVESTER program suggested that the optimal number of K is five with two sub-clusters consisting of USA accessions, one subcluster from accessions of Kazakhstan, and two subclusters from the mixture of Kazakhstan and USA accessions (S4 Fig).

### Marker-trait associations identified in six environments

**Fig 5:**
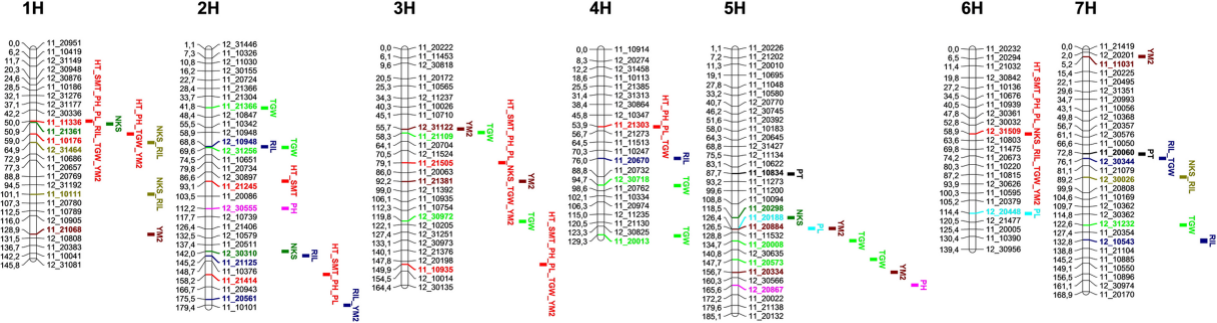
Locations of significant MTAs on seven barley chromosomes. SNPs and abbreviations of traits are given on right, and positions of SNPs are shown in cM on the left. HT–days to heading, SMT–days to seed maturation, PH–plant height, PL–peduncle length, PT–productive tillering, SL–spike length, NKS–number of kernels per spike, RIL–rachis internode length, TGW–thousand grain weight, YM2 –yield per square meter.

The LD decay curves were calculated for each chromosome and the average LD over all chromosomes at the threshold *r*^*2*^ = 0.1 was 6.8 cM (S5 Fig). The distribution lines in the QQ plots for the GWAS over eighteen field trials indicated the successful correction of the analyses due to both K and Q matrices (S6 Fig). Initially, after application of the criteria P<10E-4, 473 MTA were identified for ten traits scored in 18 field trials (6 environments x 3 years). However, only 91 MTA were statistically significant in two or more environments for nine traits, and are presented in Table 3 and Fig 5. Therefore, only those stable MTA were selected for further evaluation. Among six environments, the highest number of MTA were identified at AL (n = 65 MTA), followed by KV (n = 40) and KB (n = 39). The analyses of three plant adaptation traits HT, SMT, and PH allowed the identification of 28 MTA. HT analysis identified 9 MTA (including 8 with known chromosomal positions). In the data from SMT 8 MTA were discovered (6 MTA with known positions), and 11 MTA at PH (9 MTA with known positions). Altogether, 23 MTA with known chromosomal positions were linked to 10 SNP markers (Fig 5), and 8 of those SNPs were involved in MTA with two or three traits simultaneously. When those 7 SNPs were ignored, only two MTA for PH were identified on 2H (at 112.2 cM) and 5H (at 165.6 cM).

**Table 3:** The list of MTA identified for nine traits scored at six different stations in Kazakhstan, 2009–2011.

Trait	Marker ID	Chr.	Pos.(cM)	Pos.(bp)^1^	Alleles	MAF	R2 (%)	Min. P-value	Env.^2^
HT	11_11336	1H	50.00	261773377	G/A	0.165	4.75	9.5863E-6	4
HT	11_10176	1H	59.01	420656713	G/C	0.188	3.61	3.0892E-4	2
HT	12_10936	2H	93.14	659264767	A/G	0.114	7.62	7.3163E-5	2
HT	11_21414	2H	158.15	761624420	G/A	0.187	3.46	4.9025E-4	3
HT	11_21505	3H	79.13	580635994	G/A	0.261	9.69	4.7357E-9	7
HT	11_10935	3H	149.85	678512385	A/C	0.278	10.05	3.012E-9	7
HT	11_21303	4H	53.87	464028169	A/G	0.165	6.08	3.4229E-6	4
HT	12_31509	6H	58.91	203509034	A/G	0.264	7.59	5.9378E-8	6
HT	11_20971	U(1H)^1^	-	496660040	G/A	0.272	9.04	1.6782E-8	6
SMT	11_11336	1H	50.00	261773377	G/A	0.165	6.53	1.6693E-6	2
SMT	12_10936	2H	93.14	659264767	A/G	0.114	6.28	6.3806E-4	2
SMT	11_21414	2H	158.15	761624420	G/A	0.187	4.21	1.6473E-4	2
SMT	11_21505	3H	79.13	580635994	G/A	0.261	12.41	6.9242E-11	4
SMT	11_10935	3H	149.85	678512385	A/C	0.278	12.08	1.8496E-10	4
SMT	12_31509	6H	58.91	203509034	A/G	0.264	9.01	2.1164E-8	3
SMT	11_20971	U(1H)^1^	-	496660040	G/A	0.272	9.23	2.5175E-8	3
SMT	11_21103	U(7H)^1^	-	582767743	G/A	0.245	6.20	2.8911E-6	2
NKS	11_21361	1H	50.86	380928690	G/C	0.063	5.58	5.5684E-5	2
NKS	12_31464	1H	64.93	459030191	T/A	0.053	10.56	3.9995E-7	4
NKS	11_10111	1H	101.05	520009568	A/G	0.123	7.68	2.8751E-4	2
NKS	12_30310	2H	142.03	717373184	A/G	0.178	6.08	2.6343E-4	2
NKS	11_21505	3H	79.13	580635994	G/A	0.261	4.34	8.1255E-5	3
NKS	11_20298	5H	118.55	594971772	A/G	0.493	6.80	2.7602E-4	3
NKS	12_31509	6H	58.91	203509034	A/G	0.264	9.05	2.1404E-5	2
NKS	12_30026	7H	89.15	560756345	G/A	0.050	3.83	8.6449E-9	5
NKS	11_21103	U(7H)^1^	-	582767743	G/A	0.245	11.93	6.3414E-4	2
PH	11_11336	1H	50.00	261773377	G/A	0.165	10.38	1.4562E-9	5
PH	11_10176	1H	59.01	420656713	G/C	0.188	5.44	1.0552E-5	3
PH	12_30555	2H	112.22	691870667	A/G	0.211	3.40	4.122E-5	2
PH	11_21414	2H	158.15	761624420	G/A	0.187	4.24	1.1675E-4	2
PH	11_21505	3H	79.13	580635994	G/A	0.261	10.19	2.0175E-9	6
PH	11_10935	3H	149.85	678512385	A/C	0.278	16.21	1.8232E-13	7
PH	11_21303	4H	53.87	464028169	A/G	0.165	8.52	5.8437E-6	2
PH	12_20867	5H	165.57	648513686	G/A	0.094	3.94	1.3995E-4	2
PH	12_31509	6H	58.91	203509034	A/G	0.264	13.57	6.587E-12	3
PH	11_20971	U(1H)^1^	-	496660040	G/A	0.272	16.23	2.7324E-13	7
PH	11_21103	U(7H)^1^	-	582767743	G/A	0.245	10.47	1.2507E-9	3
PL	11_11336	1H	50.00	261773377	G/A	0.165	3.32	5.3153E-4	2
PL	11_21414	2H	158.15	761624420	G/A	0.187	4.40	5.6481E-4	2
PL	11_21505	3H	79.13	580635994	G/A	0.261	5.97	3.9028E-6	3
PL	11_10935	3H	149.85	678512385	A/C	0.278	8.17	8.556E-8	4
PL	11_21303	4H	53.87	464028169	A/G	0.165	4.82	3.9353E-5	2
PL	11_20188	5H	126.39	599123281	G/C	0.456	6.09	8.2891E-5	2
PL	12_31509	6H	58.91	203509034	A/G	0.264	9.83	4.3727E-9	4
PL	12_20448	6H	114.41	563029963	G/A	0.071	3.11	2.021E-4	2
PL	11_20971	U(1H)^1^	-	496660040	G/A	0.272	9.30	1.6741E-8	3
PL	11_21103	U(7H)^1^	-	582767743	G/A	0.245	8.83	2.4632E-8	2
PT	11_10834	5H	87.71	559204073	G/A	0.465	5.08	3.8759E-5	2
PT	11_20060	7H	72.84	109656682	C/A	0.248	6.58	3.9674E-5	2
RIL	11_11336	1H	50.00	261773377	G/A	0.165	4.84	6.1031E-5	3
RIL	12_31464	1H	64.93	459030191	T/A	0.053	3.65	4.8427E-5	4
RIL	11_10111	1H	101.05	476365128	A/G	0.123	6.19	8.2778E-5	2
RIL	12_10948	2H	68.80	183518398	G/A	0.142	8.08	9.4744E-6	2
RIL	11_21125	2H	145.20	722486571	C/G	0.071	6.80	6.7056E-4	2
RIL	11_20561	2H	175.48	754535230	A/G	0.081	6.98	7.7685E-5	2
RIL	11_20670	4H	76.01	595105468	C/G	0.071	7.06	5.7809E-5	2
RIL	12_31509	6H	58.91	203509034	A/G	0.264	3.69	5.3813E-4	3
RIL	12_30344	7H	76.06	180474019	A/G	0.068	8.89	1.5438E-4	3
RIL	12_30026	7H	89.15	560756345	G/A	0.050	12.08	4.1225E-7	2
RIL	12_10543	7H	132.76	626516365	A/G	0.095	3.47	4.4857E-4	2
TGW	11_11336	1H	50.00	261773377	G/A	0.165	4.07	1.9319E-12	3
TGW	11_10176	1H	59.01	420656713	G/C	0.188	6.41	3.1655E-6	2
TGW	11_21366	2H	41.77	34201760	G/A	0.434	3.66	3.7386E-4	3
TGW	12_31256	2H	69.55	545218326	A/C	0.076	3.22	4.2415E-6	3
TGW	11_21109	3H	58.31	261773377	G/A	0.165	4.85	2.2501E-4	2
TGW	11_21505	3H	79.13	420656713	G/C	0.188	4.51	4.244E-14	4
TGW	12_30972	3H	119.77	641853254	C/A	0.060	3.92	1.2903E-5	3
TGW	11_10935	3H	149.85	678512385	A/C	0.278	11.92	1.4975E-16	3
TGW	11_21303	4H	53.87	464028169	A/G	0.165	2.94	1.7123E-5	3
TGW	12_30718	4H	94.74	616244602	C/A	0.082	4.91	8.3033E-5	2
TGW	11_20013	4H	129.27	643005841	A/G	0.059	3.59	7.0236E-4	2
TGW	11_20008	5H	134.67	612229115	G/A	0.158	8.78	3.2776E-7	3
TGW	11_20573	5H	147.70	626279780	G/A	0.052	5.08	4.1603E-5	2
TGW	12_31509	6H	58.91	203509034	A/G	0.264	10.81	9.6308E-19	3
TGW	12_30344	7H	76.06	180474019	A/G	0.068	6.52	3.6856E-6	3
TGW	12_31232	7H	122.60	623419597	A/G	0.134	4.60	3.2969E-4	2
TGW	11_20971	U(1H)^1^	-	496660040	G/A	0.272	7.99	1.3643E-14	3
TGW	11_21103	U(7H)^1^	-	582767743	G/A	0.245	10.19	3.3725E-16	2
YM2	11_11336	1H	50.00	261773377	G/A	0.165	11.11	5.4352E-10	2
YM2	11_10176	1H	59.01	420656713	G/C	0.188	3.25	1.1038E-4	2
YM2	11_21068	1H	128.92	540586570	G/A	0.471	4.45	2.4539E-4	2
YM2	11_20561	2H	175.48	754535230	A/G	0.081	3.69	5.1955E-5	2
YM2	12_31122	3H	55.67	81047480	A/G	0.449	4.58	9.2788E-5	2
YM2	11_21505	3H	79.13	580635994	G/A	0.261	5.53	2.3481E-14	2
YM2	11_21381	3H	92.18	608636473	C/G	0.098	4.12	1.2961E-4	2
YM2	11_10935	3H	149.85	580635994	G/A	0.261	4.05	5.5605E-14	3
YM2	11_20884	5H	126.39	580635994	G/A	0.261	6.36	3.5988E-6	3
YM2	11_20334	5H	156.70	640334690	C/G	0.086	9.58	4.425E-5	3
YM2	12_31509	6H	58.91	203509034	A/G	0.264	5.66	1.6407E-12	4
YM2	11_11031	7H	5.21	8172607	G/A	0.110	4.95	3.1005E-5	2
YM2	11_21103	U(7H)^1^	-	582767743	G/A	0.245	4.40	5.2503E-11	2

^1^ –Positions are given according to the physical map Morex x Barke 2016, IBSC, GBS [42].

^2^ –Number of environments representing identified MTAs.

The GWAS of remaining traits included PT, PL, RIL, NKS, TGW, and YM2. In total 63 MTA for those six traits were identified, but only 31 MTA remained after 8 SNPs associated with HT and SMT were ignored in the analysis (Table 3 and Fig 5). After removal from further analyses of those SNPs associated with HT and SMT, the largest numbers of MTA were identified for TGW (n = 9) and RIL (n = 9). However, a number of single SNPs for RIL were shared with NKS (chromosomes 1H and 7H), with TGW (chromosome 7H), and YM2 (chromosome 5H). Nine MTA for TGW, spread over five chromosomes (2H, 3H, 4H, 5H, and 7H), with the SNP, 11_20008, on chromosome 5H (at 134.7 cM) identified with lowest *p* value. Nine MTA for RIL were detected on four chromosomes (1H, 2H, 4H, and 7H), and the list of mapped SNPs also included 12_31464 (1H, at 64.9 cM), which was significant in four environments for NKS. However, the SNP 12_10948 (2H, 68.8 cM) showed the highest significance (P<9.4744E-6) for markers identified among associations for RIL. Six MTA for NKS were mapped on four chromosomes (1H, 2H, 5H, and 7H), with the SNP 12_30026 on chromosome 7H (at 89.2 cM) identified as most significant one. However, the MTA found at the KB station (North Kazakhstan), which is the most important barley growing area in the country, were positioned on chromosome 1H (at 50.9 cM, 64.9 cM, and 101.1 cM). Two MTA for PT (chromosomes 5H and 7H) and PL (chromosomes 5H and 6H) were identified with minor significance (Table 3). Overall, 41 SNP markers with known chromosomal positions were involved in 80 associations, and 61 of them were in putative genic positions (Table 3).

## Discussion

### Field performance of the USA barley accessions in Kazakhstan

Field performance of the USA accessions in all the regions of Kazakhstan was high as the average YM2 of USA samples was only slightly less than the local accessions (Fig 2). The high field performance of USA accessions was particularly evident at the KB breeding station (Fig 3), in the Northern region of the country, where over 80% of the total barley area is cultivated annually. The assessment of key yield components, such as NKS and TGW, is clearly demonstrating that selected USA accessions can be successfully incorporated in breeding schemes for higher grain production.

As the ANOVA indicated a significant contribution of G and GE in GEI for most of the agronomic traits (S4 Table), it was important to understand patterns of these contributions in each particular case using the GGE biplot method. The application of this method has allowed not only assessment of the differences among environments, but also suggested the best breeding germplasm for different locations. For instance, AL in the HT biplot and AK in the PH analysis were well separated from the rest of the locations (S2 Fig). More separated groups were found for YM2, as locations were divided into five environments (S3 Fig). Biplots both for plant growth traits and yield components showed the split of Kazakh and USA germplasm in the majority of cases. At the same time, there were found a number of promising relationships between USA groups of samples and local environments. For instance, the genotypes of AB origin closely co-located with most of the local environments in the HT biplot, the genotypes of UT with the South Kazakhstan region in the SMT biplot, and AB samples were well aligned with the AK region (West Kazakhstan) in the PH biplot (S2 Fig). Larger prospective relationships were determined in biplots for yield components, as samples with a UT origin were possibly advantageous for the majority of environments in NKS; and germplasm of MT and WA origins for the KA station in Central Kazakhstan (S3 Fig).

### Genetic relationship of Kazakh and USA barley accessions based on SNP analysis

Despite the split of the Kazakh and the USA accessions in the majority of GGE biplots, the PCoA based on the 9K SNP analysis suggested that among the eight groups studied (166 accessions) these separated according to their geographic locations, where the set of local accessions was next to the North American set (Fig 4A), which consisted of nineteen USA and one Canadian accessions. Kazakhstan and North American groups of accessions were positioned together in the low right hand side of the plot but clearly separated from each other. Additional PCoA using 366 accessions from six Kazakh and five USA breeding origins and 2135 polymorphic SNP allowed the separation of American samples with different breeding origin into three groups. UT samples in the high right hand side of the plot were distant from BA and AB samples in low right hand side, and from MT and WA samples in the middle left hand side (Fig 4B). It is interesting that MT and WA samples plotted together with six groups of Kazakh accessions suggesting a close genetic relationship among these groups of accessions. This finding is congruent with conclusions from our study in hexaploid wheat, where Russian and Kazakh accessions were clustered together with USA accessions in a phylogenetic tree based on the Illumina SNP array analysis [43]. As the majority of Kazakh cultivars were developed in collaboration with Russian breeders by using genetic resources with Russian origin [4, 5], it can be speculated that barley breeding programs in MT and WA possibly also used germplasm sources from Russia. Alternatively, Russian, Kazakh, Montana and Washington all utilized genetic resources collected by N.I. Vavilov and H. Harlan to produce germplasm well-adapted to roughly 40^o^ N latitude [44, 45]. The high yield performance of selected accessions from MT and WA in Northern Kazakhstan (Fig 3) coincides with the outcome from the genetic relationship based on the SNP analysis.

### Identification of MTA expressed in trials of Kazakh and USA barley accessions in six regions of Kazakhstan

For plant adaptability related traits the largest number of associated SNPs mapped was identified for PH (n = 11) and HT (n = 9) associations (Table 3 and Fig 5). Also, two SNP markers (11_20971 and 11_21103) were associated with three MTA (two for PH and one for HT), not yet assigned to any chromosome. Flowering time in barley is controlled by several major genes, including vernalization, photoperiod, and independent *eps* (*EARLINESS PER SE*) and *eam* (*EARLY MATURITY*) genes [46, 47, 48]. It was particularly interesting to compare these results with those found in the work of Alqudah and co-authors (2014) [48]. In that report the majority of flowering time related genes were positioned on barley chromosomes, and QTL for different stages of flowering time were mapped separately for photoperiod sensitive, and less sensitive accessions. In this study, the assessment of MTA locations suggests that two SNPs on chromosome 1H (11_11336 and 11_10176), two SNPs on chromosome 3H (11_21505 and 11_21414), one SNP on chromosome 4H (11_21303) and one SNP on chromosome 6H (12_31509) had large pleiotropic effects, and affected at least one spike related yield components (Fig 5). It is interesting that the location of the SNP 11_21303 (4H) coincides with the location of *HvCO16 (CONSTANS) / HvPRR59 (PSEUDO-RESPONSE REGULATOR) / HvPRR73*, and the SNP 12_31509 (6H) with *HvCO5/HvPRR1/HvTOC1 (TIMING OF CAB)* shown in Alqudah et al., 2014 [48]. One of the two MTA for HT on 3H (11_10935) was mapped in close vicinity to *HvLUX* (*LUX ARRHYTHMO*) [48], but the other one (11_21505) did not match the locations of any known genes controlling flowering time. Two MTA on chromosome 1H (11_11336 and 11_10176) were located near to genes *HvCMF10* and *HvCMF11 (CCT MOTIF FAMILY*) [48], respectively. Notably, six out of eight MTA for HT identified in this study matched the positions of QTL for tipping (on chromosomes 4H and 6H) and heading phases (on chromosomes 1H, 2H, 3H and 4H) identified for photoperiod sensitive accessions in Alqudah et al. (2005) [48]. Thus, these examples are good indications that the links between markers and traits identified here have significant positive associations. For PH, six of nine MTA with known chromosomal positions were association with HT (Table 3 and Fig 5), and not directly linked to the height of plants. The positions of the remaining three MTA on chromosomes 2H, 4H, and 5H were similar to those published in Rode et al (2012) [49], Pasam et al (2012) [29], and Beheshtizadeh et al (2018) [50], respectively.

The largest number of mapped MTA (n = 18) was identified in the TGW analysis, as SNP markers for this trait were found on all chromosomes (Table 3 and Fig 5). However, when those SNPs that are also associated with HT were excluded due to pleiotropic effects, only ten MTA were found to be significant for this trait (Fig 5). The majority of these ten MTA for TGW were similarly positioned to those previously detected in European studies [23, 29], however, two MTA on chromosome 4H (12_30718 and 11_20013) can, potentially, be newly detected associations in barley.

NKS is one of the most important yield components, and it was positively correlated with YM2 in this study (Table 2). Overall, nine MTA were identified in the NKS analysis, and six of them were remained after removing three MTA that were also associated with HT. Likewise for TGW, most of the QTL with similar positions had already been identified previously in European trials [23]. However, the SNP (11_20298) on chromosome 5H (at 118.6 cM) is possibly a candidate DNA marker for a novel QTL for NKS.

The Pearson correlation index showed that NKS was negatively correlated with RIL (Table 2). A survey of the literature is suggesting that there are several known genetic factors affecting RIL, or spike density, including *uzu* (dwarfing gene) (3H) [51, 52], *Zeo1* (zeocin resistance gene) (2H) [53, 54], and *dsp1* (dense spike 1 gene) (7H) [52]. Also it has been reported that the control of RIL can be possible via linkage between a QTL for RIL and *cly1* (cleistogamy) gene on chromosome 2H [33], and directly related to variation in *HvAP2* [55, 56]. In this study, eleven MTA were identified for RIL, including four MTA found in the NKS, two in TGW, and two in HT analyses (Table 3 and Fig 5). The SNP positions in remaining three MTA for RIL did not match the genetic locations of listed genetic factors. Nevertheless, the SNP position for MTA on chromosome 2H (11_21125, at 145.2 cM) was mapped in close proximity to the QTL for RIL (at 152.9 cM) linked to *cly1* [33, 57], and the SNP positions on chromosome 7H (at 76.1 cM and at 89.2 cM) were in close vicinity to the gene *dsp1* (82.0–84.0 cM) [52]. As the chromosomal positions of genetic factors in different mapping projects can vary, those SNPs may still potentially be linked to *HvAP2* and *dsp1*. An additional study to clarify this relationship is required.

Results obtained in this study can be incorporated into local breeding programs using two ways. Firstly, a number of promising USA accessions, particularly high yield MT and WA lines in Northern Kazakhstan, will be used in crosses with local standard cultivars. Secondly, identified SNPs for MTA of studied traits will be transformed to cost effective kompetitive allele-specific PCR (KASP) assays [58]. Further, KASP assays will be validated for their efficiency in breeding projects using hybrid lines from the crosses of the USA and Kazakhstan barley lines.

## Conclusions

The performance of the USA barley accessions in field trials in six different regions of Kazakhstan was high as their average yield was not significantly less than the average yield of local accessions. In particular, the study allowed the identification of several accessions from MT, WA, AB, and BA, which outperformed the local standard cultivar in Northern Kazakhstan, where more than 90% of the barley acreage is planted. The variation in grain yield can be explained by the sensitivity of genotypes to environmental factors at crucial growth phases such as flowering time [31]. Therefore, the application of molecular markers in understanding genotype-environment interactions, and their use in early stages in breeding projects can be very efficient [59]. In this study GWAS suggested that six MTA for HT, including two on chromosome 1H, two on chromosome 3H, and one each on chromosomes 4H and 6H, have large pleiotropic effects and could be useful for improving barley grain yield potential. It is interesting that two MTA for HT on 1H (11_11336 and 11_10176) were matched the positions of genes *HvCMF10* and *HvCMF11*, and one of the two MTA on 3H (11_10935) was mapped in close vicinity to *HvLUX* [48]. The MTA on 4H (11_21303) is located close to the *HvCO16/HvPRR59/HVPRR73* genes, and the MTA on 6H is coincident with the location of the *HvCO5/HvPRR1/HvTOC1* gene cluster reported in Alqudah et al., 2014 [48]. However, the remaining MTA on 3H (11_21505) did not match the locations of any previously known major flowering genes. In addition, the MTA for HT on chromosome 2H (at 158.2 cM, 12_21414) was presumably novel association in barley identified in this study. As the effects of climate change become more obvious, international exchange and evaluation of germplasm will help ameliorate the yield penalties that local environmental changes exact.

## Supporting information

S1 Fig Geographical locations and meteorological data for the six experimental sites. KB–Karabalyk breeding station (North Kazakhstan), AK–Aktobe breeding station (West Kazakhstan), KA–Karaganda breeding station (Central Kazakhstan), KO–Kazakh Research Institute of Rice (Kyzylorda city, South Kazakhstan), KV–Krasnovodopad breeding station (South Kazakhstan), AL–Almaty breeding station (South-east Kazakhstan). **(**A) The locations of the six field trials. (B) The average precipitation (mm) at the six sites for the years 2009–2011. (C) The average daylength (hours) for each day of the month at the six sites in 2009–2011. (D) The average mean temperature data (C^0^) at the six sites in 2009–2011.
(TIF)

S2 Fig GGE biplot analysis for adaptive traits for the barley accessions from five USA and six Kazakh breeding organizations grown at six sites in Kazakhstan. KB–Karabalyk breeding station (North Kazakhstan), AK–Aktobe breeding station (West Kazakhstan), KA–Karaganda breeding station (Central Kazakhstan), KO–Kazakh Research Institute of Rice (Kyzylorda city, South Kazakhstan), KV–Krasnovodopad breeding station (South Kazakhstan), AL–Almaty breeding station (South-east Kazakhstan), MT–Montana State University, WA–Washington State University, UT–Utah State University, AB–Small Cereal Collection of the USDA held in Aberdeen (Idaho), BA–Busch Agricultural Resources, a division of the Anheuser-Busch Corporation. Graphs were constructed based on the normalized scatter plot method. PC1 and PC2 are Principal Coordinates of the analyses. (A) The plot for heading time. (B) The plot for seed maturation time. (C) The plot for plant height.
(TIF)

S3 Fig GGE biplot analysis for grain yield related traits for barley accessions from five USA and six Kazakh breeding organizations grown at six sites in Kazakhstan. KB–Karabalyk breeding station (North Kazakhstan), AK–Aktobe breeding station (West Kazakhstan), KA–Karaganda breeding station (Central Kazakhstan), KO–Kazakh Research Institute of Rice (Kyzylorda city, South Kazakhstan), KV–Krasnovodopad breeding station (South Kazakhstan), AL–Almaty breeding station (South-east Kazakhstan), MT–Montana State University, WA–Washington State University, UT–Utah State University, AB–Small Cereal Collection of the USDA held in Aberdeen (Idaho), BA–Busch Agricultural Resources, a division of the Anheuser-Busch Corporation. Presented graphs developed based on normalized scatter plot method. PC1 and PC2 are Principal Coordinates of the analyses. (A) The plot for number of kernels per spike. (B) The plot for thousand grains weight. (C) The plot for yield per square meter.
(TIF)

S4 Fig Genetic differentiation of 380 two-rowed spring barley accessions using 2135 SNP markers. Clustering of samples was done using the STRUCTURE software.
(TIF)

S5 Fig LD decay line (threshold *r*^2^ = 0.1) for whole barley genome based on the analysis of 366 barley accessions and 2135 polymorphic SNP markers. (TIF)

S6 Fig QQ plots for number of kernels per spike trait at Karaganda breeding station (2011) by using GLM and MLM methods in TASSEL 5.0 package. (A) QQ plot by using GLM. (B) QQ plot by using GLM + Q matrix. (C) QQ plot by using MLM + K matrix. (D) QQ plot by using MLM + K + Q matrices.
(TIF)

S1 Table List of two-rowed spring accessions from six breeding programs in Kazakhstan. (XLS)

S2 Table List of two-rowed spring barley accessions from five breeding programs in the USA. (XLS)

S3 Table List of barley accessions from 8 regions of the World genotyped using 9K SNP Illumina array. (XLS)

S4 Table Three-way ANOVA for eight studied traits of two-rowed spring barley accessions across eighteen environments. (XLS)
