# Supplementary figures and images for: Marker-trait associations in two-rowed spring barley accessions from Kazakhstan and the USA
Source: PLoS One. 2018 Oct 11;13(10):e0205421. doi: 10.1371/journal.pone.0205421 (PMC6181366; doi:10.1371/journal.pone.0205421)

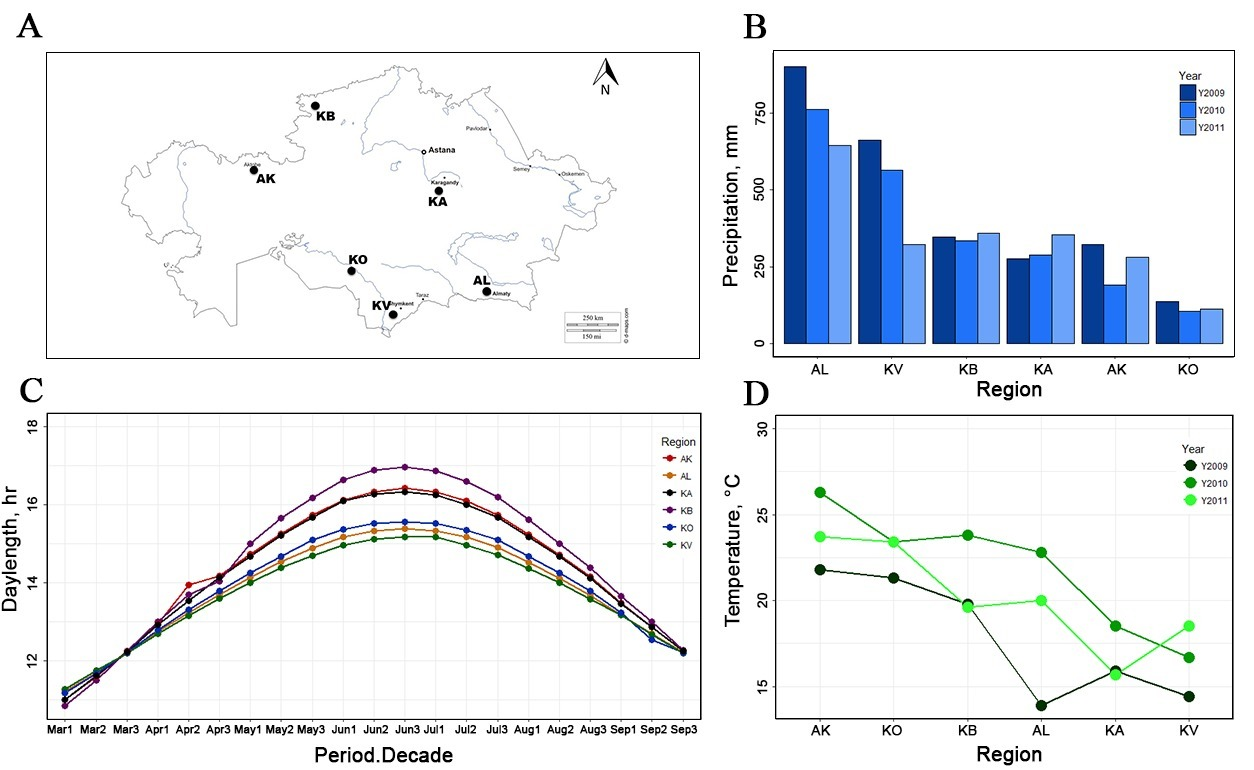

Supplement: S1 Fig — KB–Karabalyk breeding station (North Kazakhstan), AK–Aktobe breeding station (West Kazakhstan), KA–Karaganda breeding station (Central Kazakhstan), KO–Kazakh Research Institute of Rice (Kyzylorda city, South Kazakhstan), KV–Krasnovodopad breeding station (South Kazakhstan), AL–Almaty breeding station (South-east Kazakhstan). (A) The locations of the six field trials. (B) The average precipitation (mm) at the six sites for the years 2009–2011. (C) The average daylength (hours) for each day of the month at the six sites in 2009–2011. (D) The average mean temperature data (C0) at the six sites in 2009–2011. (TIF) [file pone.0205421.s001.tif]

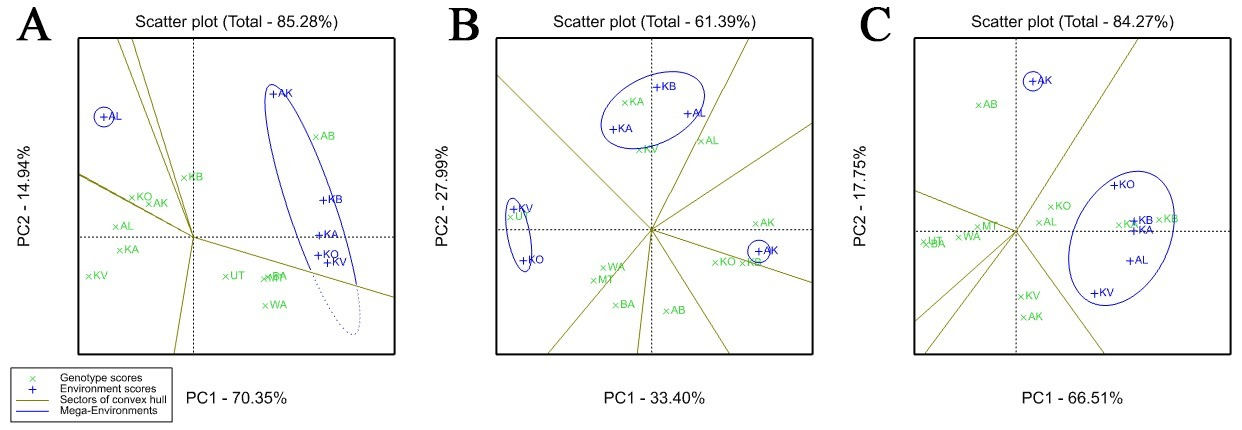

Supplement: S2 Fig — KB–Karabalyk breeding station (North Kazakhstan), AK–Aktobe breeding station (West Kazakhstan), KA–Karaganda breeding station (Central Kazakhstan), KO–Kazakh Research Institute of Rice (Kyzylorda city, South Kazakhstan), KV–Krasnovodopad breeding station (South Kazakhstan), AL–Almaty breeding station (South-east Kazakhstan), MT–Montana State University, WA–Washington State University, UT–Utah State University, AB–Small Cereal Collection of the USDA held in Aberdeen (Idaho), BA–Busch Agricultural Resources, a division of the Anheuser-Busch Corporation. Graphs were constructed based on the normalized scatter plot method. PC1 and PC2 are Principal Coordinates of the analyses. (A) The plot for heading time. (B) The plot for seed maturation time. (C) The plot for plant height. (TIF) [file pone.0205421.s002.tif]

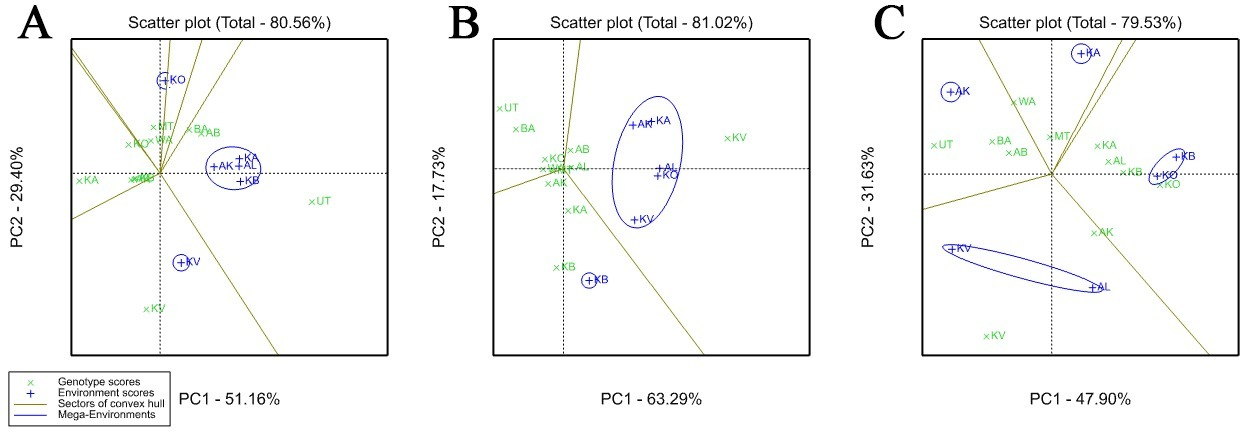

Supplement: S3 Fig — KB–Karabalyk breeding station (North Kazakhstan), AK–Aktobe breeding station (West Kazakhstan), KA–Karaganda breeding station (Central Kazakhstan), KO–Kazakh Research Institute of Rice (Kyzylorda city, South Kazakhstan), KV–Krasnovodopad breeding station (South Kazakhstan), AL–Almaty breeding station (South-east Kazakhstan), MT–Montana State University, WA–Washington State University, UT–Utah State University, AB–Small Cereal Collection of the USDA held in Aberdeen (Idaho), BA–Busch Agricultural Resources, a division of the Anheuser-Busch Corporation. Presented graphs developed based on normalized scatter plot method. PC1 and PC2 are Principal Coordinates of the analyses. (A) The plot for number of kernels per spike. (B) The plot for thousand grains weight. (C) The plot for yield per square meter. (TIF) [file pone.0205421.s003.tif]

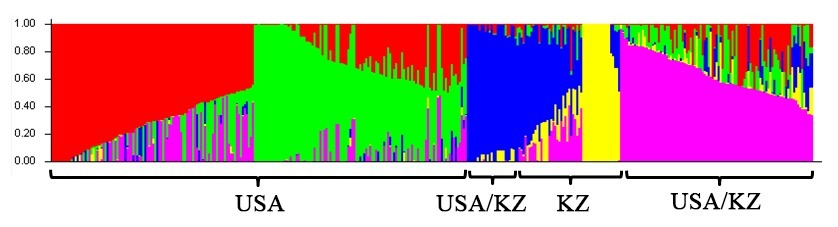

Supplement: S4 Fig — Clustering of samples was done using the STRUCTURE software. (TIF) [file pone.0205421.s004.tif]

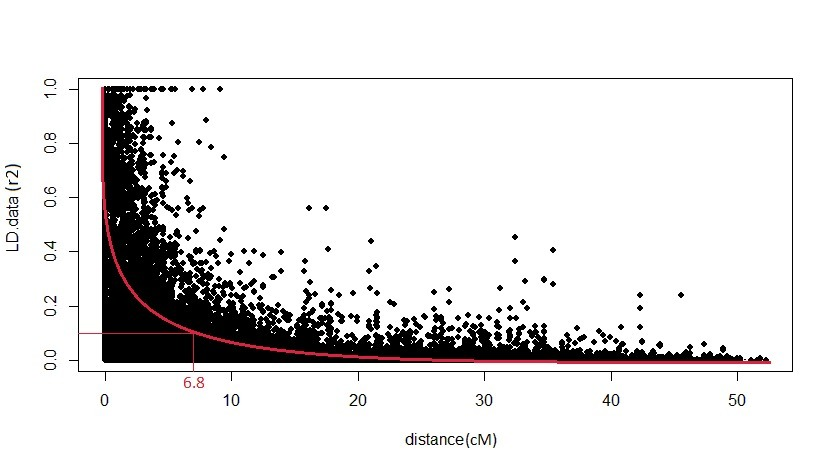

Supplement: S5 Fig — (TIF) [file pone.0205421.s005.tif]

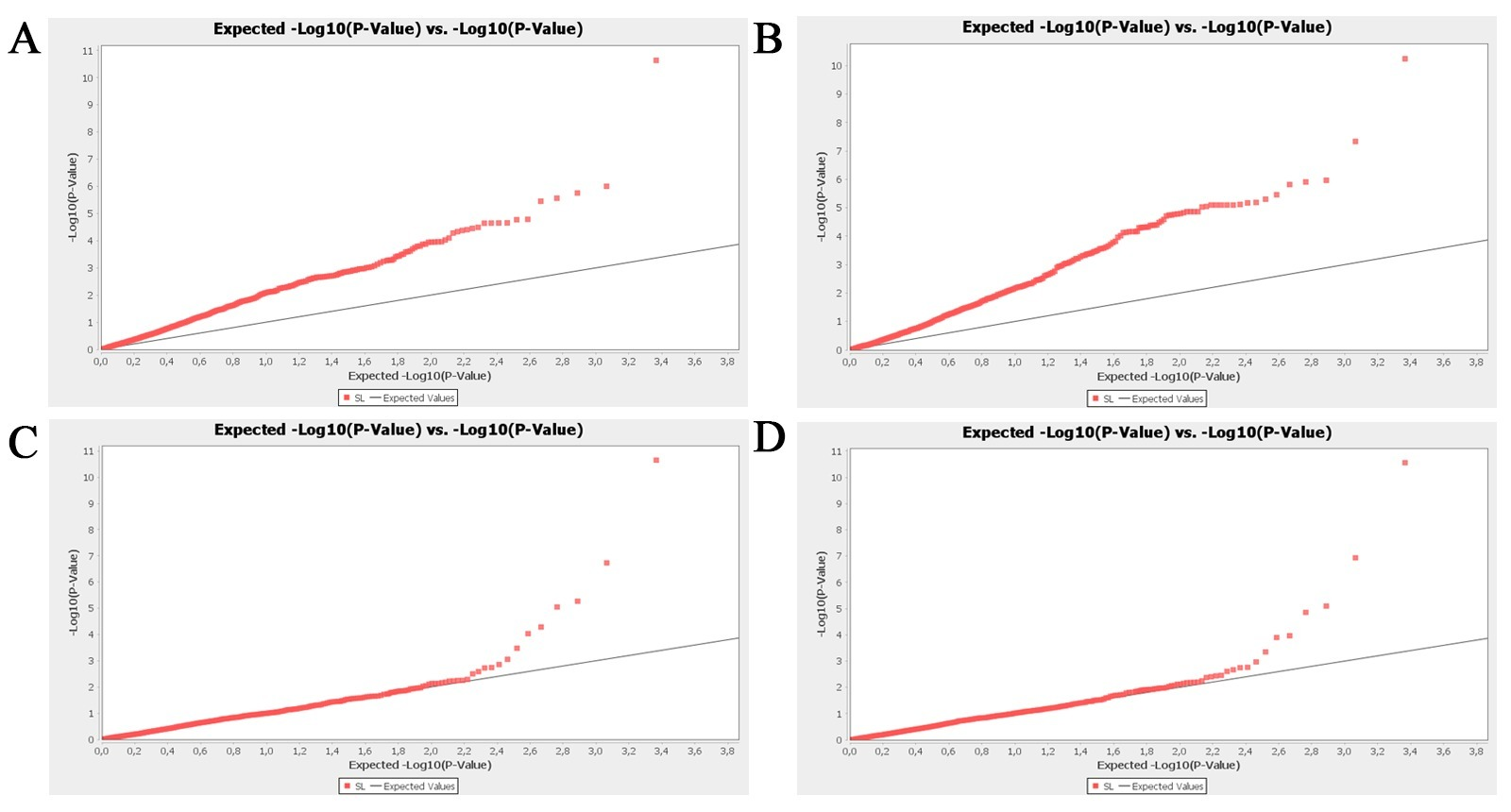

Supplement: S6 Fig — (A) QQ plot by using GLM. (B) QQ plot by using GLM + Q matrix. (C) QQ plot by using MLM + K matrix. (D) QQ plot by using MLM + K + Q matrices. (TIF) [file pone.0205421.s006.tif]
